# Precipitation Biases in CMIP5 Models over the South Asian Region

**DOI:** 10.1038/s41598-019-45907-4

**Published:** 2019-07-03

**Authors:** Raju Pathak, Sandeep Sahany, Saroj Kanta Mishra, S. K. Dash

**Affiliations:** 1 Centre for Atmospheric Sciences, Indian Institute of Technology Delhi, Delhi, India; 2 DST Centre of Excellence in Climate Modelling, Indian Institute of Technology Delhi, Delhi, India

**Keywords:** Atmospheric science, Atmospheric dynamics

## Abstract

Using data from 33 models from the CMIP5 historical and AMIP5 simulations, we have carried out a systematic analysis of biases in total precipitation and its convective and large-scale components over the south Asian region. We have used 23 years (1983–2005) of data, and have computed model biases with respect to the PERSIANN-CDR precipitation (with convective/large-scale ratio derived from TRMM 3A12). A clustering algorithm was applied on the total, convective, and large-scale precipitation biases seen in CMIP5 models to group them based on the degree of similarity in the global bias patterns. Subsequently, AMIP5 models were analyzed to conclude if the biases were primarily due to the atmospheric component or due to the oceanic component of individual models. Our analysis shows that the set of individual models falling in a given group is somewhat sensitive to the variable (total/convective/large-scale precipitation) used for clustering. Over the south Asian region, some of the convective and large-scale precipitation biases are common across groups, emphasizing that although on a global scale the bias patterns may be sufficiently different to cluster the models into different groups, regionally, it may not be true. In general, models tend to overestimate the convective component and underestimate the large-scale component over the south Asian region, although with spatially varying magnitudes depending on the model group. We find that the convective precipitation biases are largely governed by the closure and trigger assumptions used in the convection parameterization schemes used in these models, and to a lesser extent on details of the individual cloud models. Using two different methods: (i) clustering, (ii) comparing the bias patterns of models from CMIP5 with their AMIP5 counterparts, we find that, in general, the atmospheric component (and not the oceanic component through biases in SSTs and atmosphere-ocean feedbacks) plays a major role in deciding the convective and large-scale precipitation biases. However, the oceanic component has been found important for one of the convective groups in deciding the convective precipitation biases (over the maritime continent).

## Introduction

The multi-model mean (widely used in climate change projections by the Intergovernmental Panel on Climate Change) assumes inter-model statistical independence (SI)^1,2^. However, the SI assumption is not quite accurate (Pennell and Reichler^3^; Pincus *et al*.^4^). For example, Pennell and Reichler^3^ reported an effective ensemble size much smaller than the actual number of CMIP3 models. SI violations are very large in CMIP5 than CMIP3 models due to multiple reasons, especially similarities in numerical schemes, physical parameterizations, etc. (Pincus *et al*.^4^; Masson and Knutti^5^). For example, IPSL-CM5A-LR, IPSL-CM5A-MR, and IPSL-CM5B-LR were developed by slight modification in resolution and the atmospheric component. Similar is the case with GISS-E2H and GISS-E2R. GFDL-ESM2M and GFDL-ESM2G primarily differ only in their ocean components. MIROC-ESM and MIROC-ESM-CHEM differ in ocean biogeochemistry and atmospheric chemistry. MPI-ESM-LR and MPI-ESM-P were developed from their predecessor by changing resolution and neglecting the feedback between dynamic vegetation and land use.

Although sharing of components between various model versions from the same center may sound obvious, even different modeling centers share large fraction of the model code. For example, CNRM and EC-EARTH use similar atmospheric components (AEPEGE/IFS/ECMWF); ACCESS uses the HadGEM2 atmospheric component; FGOALS uses several physical parameterizations from CCSM; NorESM uses some key components of CESM1. All such similarities in codes, schemes and concepts among the various CMIP5 models have been found to be a reason for common biases in the simulated fields^5–8^. Past studies on model similarity and genealogy have reported the use of similar ocean component to be less relevant than the use of similar atmospheric component in producing large model similarity in surface climatology^5,8^. In simpler words, it means that the surface climatology from two models with similar atmospheric components but different ocean components would have greater commonality than that of two models with similar ocean components but different atmospheric components.

Common biases in the simulated fields are found to be usually large in precipitation simulation, with largest over the south Asian region during the southwest summer monsoon^9–13^. Deficiencies in precipitation simulation by models have primarily been due to the persistent errors in the simulation of location and timing, and improving the spatial and seasonal features would provide a better model agreement in historical and future^14^. Regionally, the Arabian Sea cold SST bias during the pre-monsoon season in some of the CMIP5 models has also been found to be important for the simulation of south Asian summer monsoon precipitation^15^. In the context of climate change, precipitation is expected to increase in the future due to increased human influence and anthropogenic emissions^16,17^, and thus changing the water cycle^18^. Reliable precipitation simulation over the south Asian region is crucial for society, and for mitigation and adaptation strategies due to the changes in its pattern and variability under climate change^19–21^.

There have been studies on model genealogy as discussed above, using total precipitation as the variable of interest, but common biases across models in the individual precipitation components (convective and large-scale) have never been analyzed. Evaluating model similarity in biases in precipitation components is critically important because most of the model development efforts have focused on reducing the common biases in total precipitation, leading to an invisible bias in the individual components (e.g., He *et al*.^22^).

In this paper we investigate common biases in CMIP5 models by using total precipitation as well as its convective and large-scale components, and for each of the three variables we divide the models into broad groups based on the patterns of their biases on a global scale. Subsequently, the ability of models belonging to a particular group in simulating the south Asian summer monsoon and tropical waves is evaluated. In addition, AMIP5 model results have also been used to explore if the reported biases in the corresponding models from CMIP5 behave any different with prescribed sea surface temperatures.

## Results and Discussion

**Figure 1:**
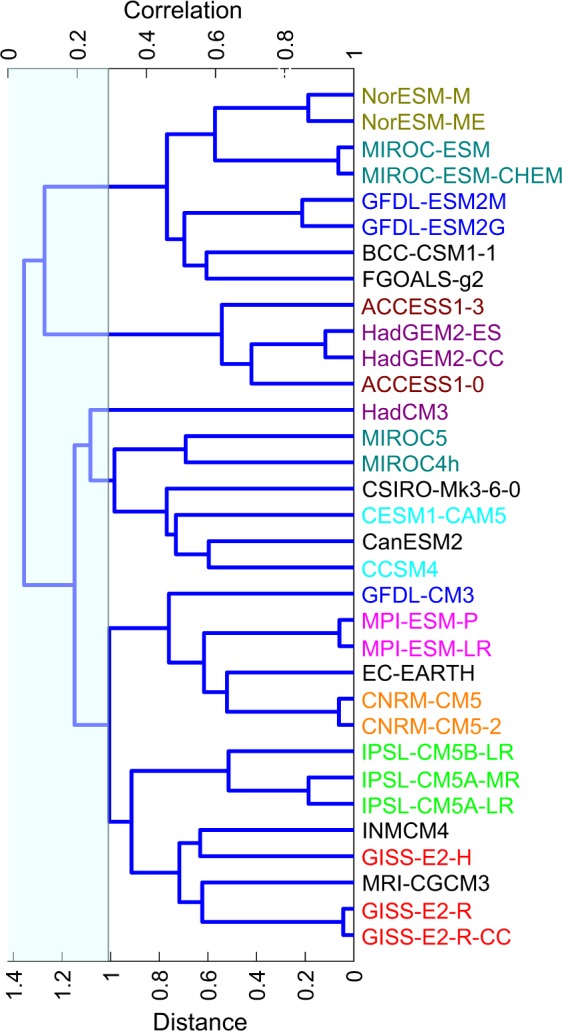
Hierarchical clustering in CMIP5 models based on the correlation in model biases for mean annual total precipitation (40S–40N; 0–360E). The clustering method is based on weighted pairwise average distance algorithm^33^. The models developed at same center/institution are shown in same color.

We have carried out a systematic analysis of similarity and dissimilarity in bias structures of CMIP5 and AMIP5 models in simulating the partitioning of precipitation between the convective and large-scale components. Figure 1 shows the hierarchical structure of CMIP5 models for total precipitation simulation. Models developed either at the same center (same color) or at different centers falling in the same branch (see Section 2 for details on methodology used for branching) show large similarity in total precipitation simulation, whereas models in the farthest branches show highest dissimilarity. From Table 1, and from Knutti *et al*.^7,8^ and Dai^19^, we found some of the obvious similarities between same center or between different center models shown in Fig. 1 are arising due to the similarities in atmospheric component in spite of having different ocean components or inclusion of ocean biogeochemistry, and atmospheric chemistry. In other words, if two climate models either from the same center or different centers have the same atmospheric component their total precipitation bias is very similar, irrespective of the other components. For example, ACCESS and HadGEM show high similarity, even though they have only the atmospheric component in common. The level of similarity is very similar to model pairs that have lot more common components than that shared between ACCESS and HadGEM. For example, the level of similarity between ACCESS and HadGEM is not very different than that between: (i) MIROC-ESM-CHEM and MIROC-ESM (the former having an additional component in the form of the atmospheric chemistry package), (ii) NorESM1-ME and NorESM1-M (the former having an additional component in the form of the ocean biogeochemistry package), (iii) GISS-E2R-CC and GISS-E2R (the former having an additional component in the form of carbon cycle package), (iv) HadGEM2-ES and HadGEM2-CC (the former having an additional component in the form of atmospheric chemistry package), and (v) GFDL-ESM2G and GFDL-ESM2M (the former having different ocean component). Figure 1 also shows large dissimilarity between model pairs that started with a similar parent atmospheric component that underwent significant modification during model development. For example, dissimilarity seen between (i) MIROC-4h/5 and MIROC-ESM/ESM-CHEM, (ii) GFDL-CM3 and GFDL-ESM2G/2M, and (iii) CCSM4 and CESM-CAM5, are due to significant modifications in the atmospheric components during the model development process.

**Table 1:** Model component Description, Resolution (latitude × longitude), Vertical level.

Models	Atmosphere, resolution, vertical level, reference	Ocean, resolution, vertical level	Country
GFDL-CM3	CM3, ~*1.802* × *1.802*, **48**, Donner [2011]	MOM4.1, *1–2*.*342* × *1.802*, **50**	USA
GFDL-ESM2G	CM2.1, *2.5* × *2*, **48**, Delworth [2006]	Gold, *1–2*.*342* × *1.802*, **50**	USA
GFDL-ESM2M	CM2.1, *2.5* × *2*, **48**, Delworth [2006]	MOM4.1, *1–2.342* × *1.802*, **50**	USA
GISS-E2R	*2* × *2.5*, **40**	Russell Ocean, *1* × *1.25*, **32**	USA
GISS-E2H	*2* × *2.5*, **40**	HYCOM Ocean, *0.2–1* × *1*, **26**	USA
GISS-E2R-CC	*~1* × *1*, **40**	Russell Ocean, *1* × *1.25*, **32**	USA
CESM-CAM5	CAM5, *0.9* × *1.25*, **27**, Neale [2010; 2013]	Modified POP2, *1.125* × *0.27–0.64*, **60**, Danabasoglu [2012]	USA
CCSM4	CAM4, *0.9* × *1.25*, **27**, Neale [2010 & 2013]	Modified POP2, *1.125* × *0.27–0.64*, **60**, Danabasoglu [2012]	USA
IPSL-CM5A-LR	LMDZ5A, *1.9* × *3.75*, **39**	*2* × *2–0.5*, **31**, Madec [2008]	France
IPSl-CM5A-MR	LMDZ5A, *1.25* × *2.5*, **39**	*2* × *2–0.5*, **31**, Madec [2008]	France
IPSL-CM5B-LR	LMDZ5B, *1.9* × *3.75*, **39**	*2* × *2–0.5*, **31**, Madec [2008]	France
CNRM-CM5	ARPEGE-Climat v5.2, (IFS), ~*TL127*, **31**, Déqué [1994] and Voldoire [2013]	NEMOv3.2, *~ 0.7*, **42**, Madec [2008]	France
CNRM-CM5–2	ARPEGE-Climat v5.2, (IFS), ~*TL127*, **31**, Déqué [1994] and Voldoire [2013]	NEMOv3.2, ~ *0.7*, **42**, Madec [2008]	France
MIROC4h	AGCM5. 7,*0.563* × *0.563*, **56**	COCO3.4, *0.28* × *0.19*, **48**, Hasumi [2000]	Japan
MIROC5	AGCM6, *1.406* × *1.406*, **40**, Nozawa [2007] and Watanabe [2008a]	COCO4.5, *1.4* × *0.5*–1.4, **50**, Hasumi [2006]	Japan
MIROC-ESM	MIROC-AGCM, *2.813* × *2.813*, **80**	COCO3.4, *1.4* × *0.5–1.4*, **44**	Japan
MIROC-ESM-CHEM	MIROC-AGCM, *2.813* × *2.813*, **80**	COCO3.4, *1.4* × *0.5–1.4*, **44**	Japan
CSIRO-Mk3-6-0	*~1.875* × *1.875*, **18**, Gordon [2002; 2010] and Rotstayn [2012]	Modified MOM2.2, *~0.9* × *1.875*, Gordon [2002 and 2010]	Australia
ACCESS1-0	HadGEM2, *1.729* × *1.306 N96*, **38**, Martin [2011], Bi [2013b] and Rashid [2013)	ACCESS-OM (MOM4p1), *~1* × *1*, **50**, Bi [2013a] and Marsland [2013]	Australia
ACCESS1-3	GAM1.0, *1.729* × *1.306 N96*, **38**, Hewitt [2011], Bi [2013b] and Rashid [2013]	ACCESS-OM (MOM4p1), *~1* × *1*, **50**, Bi [2013a] and Marsland [2013]	Australia
HadCM3	HadAM3, *3.75* × *2.5*, **19**, Pope [2000]	HadOM, *1.25* × *1.25*, **20**	UK
HadGEM2-CC	HadGEM2, *1.875* × *1.25*, **60**	*1.875* × *1.25*, **40**	UK
HadGEM2-ES	HadGEM2, *1.875* × *1.25*, **38**, Davies [2005]	*1* × *1 30NS & 1/3° at equato*r, **40**	UK
MPI-ESM-P	ECHAM6, *~1.8 T63*, **47**, Stevens [2012]	MPIOM, *~1*.*5° GR15*, **40**, Jungclaus [2013]	Germany
MPI-ESM-LR	ECHAM6, *~1.8 T63*, **47**, Stevens [2012]	MPIOM, *~1.5° GR15*, **40**, Jungclaus [2013]	Germany
NorESM1-M	CAM4-Oslo, *1.9* × *2.5*, **26**, Neale [2010] and Kirkevåg [2013]	NorESM-Ocean, *1.125 along the equator*, **53**	Norway
NorESM1-ME	CAM4-Oslo, *1.9* × *2.5*, **26**, Neale [2010] and Kirkevåg [2013]	NorESM-Ocean *1.125*, *long the equator*, **53**	Norway
BCC-CSM1-1	BCC-AGCM2.1, *T4*2 × *T42*, **L26**, Wu [2008b; 2010a, and 2012]	MOM4-L40, *~1° with 1/3 at the equator*, **40**, Griffies [2005]	China
FGOALS-g2	GAMIL2, *2.813* × *2.813*, **26**, Wang [2004] and Li [2013b]	LICOM2, *1* × *1with 0.5 at merid*. *in tropical*, **30**, Liu [2012a]	China
INM-CM4	*2* × *1.5*, **21**	*1* × *0.5*, **40**, Volodin *et al*. [2010]; Zalesny *et al*. [2010]	Russia
CanESM2	*Spectral T63*, **35**, von Salzen [2013]	*1.171* × *1.067*, **40**, Merryfield [2013]	Canada
EC-EARTH	IFS-c31r1, *~1.125* × *T159*, **L62**, Hazeleger [2012]	NEMO_ecmwf, ~*1* × *1*, Hazeleger [2012]	Europe

**Figure 2:**
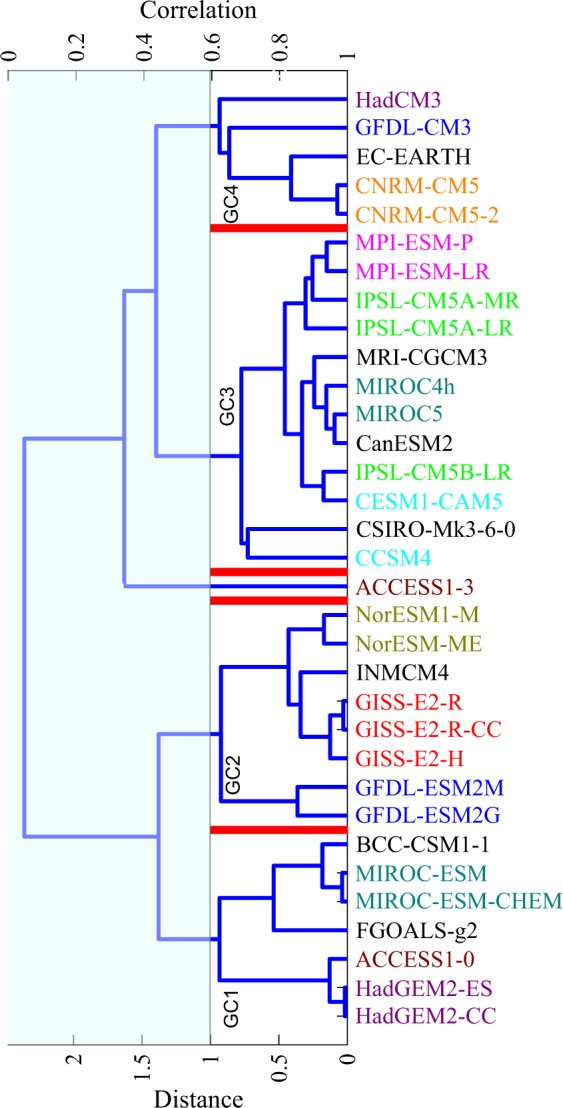
Same as Fig. 1 but with clustering done based on convectiveprecipitation.

**Figure 3:**
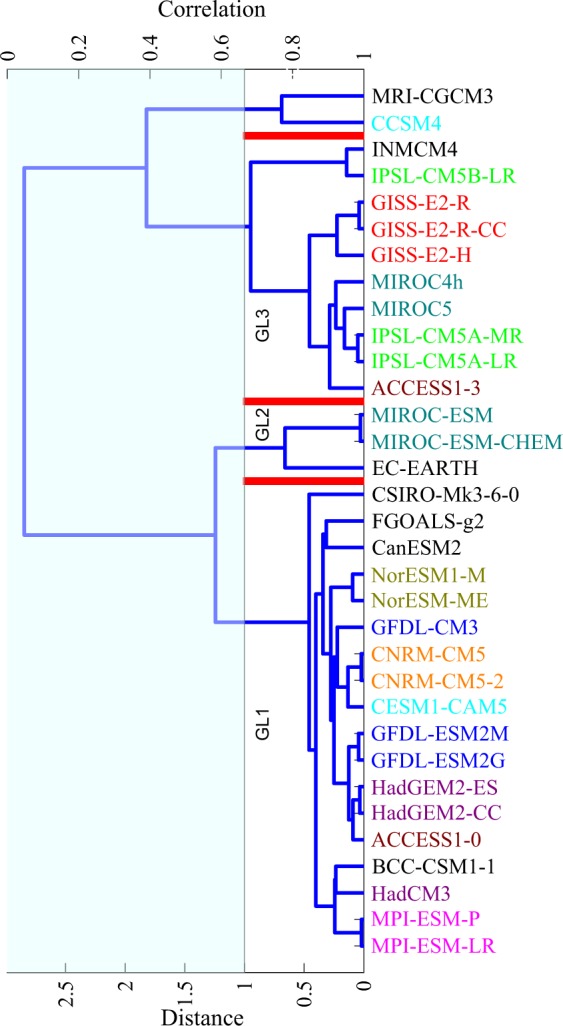
Same as Fig. 1 but with clustering done based on large-scale precipitation.

Above results on model similarity for total precipitation is consistent with findings related to CMIP3 models (e.g., Annamalai *et al*.^9^, Pincus *et al*.^4^, Bollasina *et al*.^23^), and CMIP5 models (e.g., Sperber *et al*.^11^), that state that monsoon precipitation biases in atmosphere-only models (AGCMs) are similar to the atmosphere-ocean coupled models (AOGCMs). Precipitation partitioning between convective and large-scale components are simulated separately by convective and large-scale parameterization schemes of the atmospheric component^22,24,25^. Figures 2 and 3 show the hierarchical structure of CMIP5 models based on their similarity in simulating the precipitation partitioning. As can be seen from Figs 1–3, similarity between models in simulating total precipitation (see Fig. 1) may not necessarily imply similarity in simulating the convective (see Fig. 2) and large-scale components (see Fig. 3).

### Convective and large-scale precipitation parameterizations used in CMIP5 models

Convective parameterization schemes are generally based on one of the following cloud model types: (i) Spectral cloud ensemble, similar to Arakawa and Schubert^26^, or (ii) Bulk cloud ensemble, or (iii) Combination of spectral and bulk ensemble (Zhang and McFarlane [hereafter ZM]^27^; for more details see Table 2). For example, GFDL and MIROC models are based on approach (i), GISS-E2R/H and GISS-E2R-CC, HaDGEM2-ES/CC and HadCM3, ACCESS, MPI-ESM-LR/P, CNRM-CM5/5-2, CSIRO-Mk3-6-0, and EC-EARTH models are based on approach (ii), and CCSM4, CESM-CAM5, FGOALS, and NorESM models are based on approach (iii). In addition to cloud model type, the closure and triggering mechanism of convective parameterization (for more details see Table 2) also controls the total precipitation and its partitioning. In CMIP5, most of the models use convective available potential energy (CAPE) or dilute CAPE (DCAPE) based closure and trigger function (as can be seen from Table 2), whereas, few models use moisture convergence-based closure and moisture convergence or relative humidity-based triggers. In CMIP5, most of the models use prognostic cloud condensate-based approach in their large-scale precipitation parameterization (for more details see Table 2), and hence exhibit large similarity in the simulated large-scale precipitation (discussed later).

**Table 2:** Description of Convective and Large-scale parameterization, Convective triggers and Convective closures.

Models	Convective precipitation	Large-scale precipitation	Convective Trigger	Convective Closure
**Cloud Model Type: Spectral Cloud Ensemble**				
GFDL-CM3	Relaxed Arakawa–Schubert scheme of Moorthiand Suarez [1992] with few modifications in physics from Donner *et al*. [2011]	Cloud microphysics of Rotstayn [2000] and macrophysics from Tiedtke [1993], stratiform clouds from Golaz *et al*. [2011]	Cloud work function (CWF) similar to dilute cape (DCAPE)	CAPE closure towards a threshold over a relaxation time scale
GFDL-ESM2G	Relaxed Arakawa–Schubert scheme of Moorthiand Suarez [1992] and Dunne *et al*. [2012 and 2013]	Same as GFDL-CM3	Cloud work function (CWF) similar to DCAPE	CAPE closure towards a threshold over a relaxation time scale
GFDL-ESM2M	Same as GFDL-ESM2G	Same as GFDL-CM3	Cloud work function (CWF) similar to DCAPE	CAPE closure towards a threshold over a relaxation time scale
MIROC5	Entraining plume model scheme of Chikira *et al*. [2010] similar to Gregory [2001] with some modification according Pan and Randall [1998]	Prognostic large-scale cloud scheme of Watanabe *et al*. [2009] and bulk microphysical scheme from Wilson and Ballard [1999]	CAPE	Prognostic convective kinetic energy closure similar to CAPE closure
MIROC4h	Prognostic closure Arakawa Schubert scheme from Pan and Randall [1998] and addition of relativehumidity-based suppression condition by Emori *et al*. [2001]	Prognostic cloud water scheme of Treutand Li [1991]	Relative humidity	Prognostic convective kinetic energy closure similar to CAPE closure
MIROC-ESM	Same as MIROC4h	Large-scale condensation is diagnosed based on Treut& Li (1991) and simple cloud microphysics scheme	Relative humidity	Prognostic convective kinetic energy closure similar to CAPE closure
MIROC-ESM-CHEM	Same as MIROC4h	Same as MIROC-ESM	Relative humidity	Prognostic convective kinetic energy closure similar to CAPE closure
**Cloud Model Type: Bulk Cloud Ensemble**				
GISS-E2R	Bulk mass flux scheme by Delgenio& Yao (1993)	Prognostic stratiform cloud based on moisture convergence by Delgenio *et al*. (1996)	—	Moisture convergence
GISS-E2H	Same as GISS-E2R	Same as GISS-E2R	—	Moisture convergence
GISS-E2R-CC	Same as GISS-E2R	Same as GISS-E2R	—	Moisture convergence
HadCM3	Bulk mass flux scheme by Gregory & Rowntree (1990)	Large-scale precipitation is calculated based on cloud water and ice contents similar to Smith [1990]	Cloud base buoyancy	CAPE
HadGEM2-CC	Same as HadCM3, withan additional adaptive detrainment parameterization by Derbyshire *et al*. [2011]	Same as HadCM3	Cloud base buoyancy	CAPE
HadGEM2-ES	Same as HadGEM2-CC	Same as HadCM3	Cloud base buoyancy	CAPE
ACCESS1-0	Same as HadGEM2-CC	Same as HadCM3	Cloud base buoyancy	CAPE
ACCESS1-3	Same as in ACCESS1.0, except physical parameterization, which is similar to GAM1.0	Same as HadCM3	—	CAPE
MPI-ESM-LR	Bulk mass flux scheme by Tiedtke [1989] with modifications in deep convection by Nordeng *et al*. [1994]	Prognostic equations of the water phases, bulk cloud microphysics from Lohmann andRoeckner [1996]	Moisture convergence and buoyant surface air when lifted to the LCL	Moisture convergence/adjustment type
MPI-ESM-P	Same as MPI-ESM-LR	Same as MPI-ESM-LR	Moisture convergence and buoyant surface air when lifted to the LCL	Moisture convergence/adjustment type
MRI-CGCM3	Same as MPI-ESM-LR		Moisture convergence	CAPE
CNRM-CM5	Mass-flux scheme of Bougeault [985]	Statistical cloud scheme of Ricard and Royer [1993]	Depends on moisture convergence and stability profile	Moisture convergence
CNRM-CM5-2	Same as CNRM-CM5	Same as CNRM-CM5	Depends on moisture convergence and stability profile	Moisture convergence
EC-EARTH	Bulk mass-flux scheme and Entraining/detraining plume cloud model by Hazeleger *et al*. [2010]	saturated downdraughtsandsimple microphysics scheme	—	CAPE
CSIRO-Mk3-6-0	Bulk mass flux convection scheme of Gregory and Rowntree [1990] with slightly modified by Gregory [1995]	Stratiform cloud condensate scheme from Rotstayn([000]	—	Stability-Dependent Closure
**Cloud Model Type: Mixed Nature of Spectral and Bulk Cloud Ensemble**				
CCSM4	Simplified Arakawa and Schubert cumulus ensemble scheme of Zhang and McFarlane^27^ with plume dilution of Neale *et al*. [2008]	Prognostic condensate and precipitation parameterization from Zhang *et al*. [2003]	CAPE	DCAPE
CESM-CAM5	Same as CCSM4 with few more modifications by Neale *et al*. [2012]	Same as CCSM4	CAPE	DCAPE
NorESM1-M	Same as CCSM4	Same as CCSM4	CAPE	DCAPE
NorESM1-ME	Same as CCSM4	Same as CCSM4	CAPE	DCAPE
BCC-CSM1-1	Mass flux scheme developed by Zhang and McFarlane [1995], has been adapted as proposed by Wu *et al*. [2010]	Same as CCSM4	CAPE	CAPE
FGOALS-g2	Mass flux type cumulus convection developed by Zhang and McFarlane^27^	Precipitation occurs whenever the local relative humidity is supersaturated	CAPE	CAPE
CanESM2	Mass flux type cumulus convection schemeby Scinocca and McFarlane [2004]	Prognostic cloud liquid water and ice, statistical cloud scheme, interactive with aerosols	CAPE	Cloud base closure
IPSL-CM5A-LR	Episodic mixing and buoyancy sorting scheme by Emanuel [1991] and modified moist convection scheme by Grandpeix *et al*. [2004]	Cloud cover and in-cloud water deduced from large-scale total water and moisture at saturation from Bony and Emmanuel [2001]	—	CAPE closure
IPSl-CM5A-MR	Same as IPSL-CM5A-LR	Same as IPSL-CM5A-LR	—	CAPE closure
IPSL-CM5B-LR	Same as IPSL-CM5A-LR, with the modification in closure and trigger mechanism byGrandpeix and Lafore [2010]	Same as IPSL-CM5A-LR, with the few modifications by Jam *et al*. [2011]	Available Lifting Energy	Available Lifting Power
INM-CM4	Lagged convective adjustment after Betts [1986], but modified referenced profile for deep convection	Stratiform cloud fraction is calculated as linear function of relative humidity	—	CAPE

### Model grouping based on precipitation partitioning

CMIP5 models are clustered into the various convective groups, namely, GC1, GC2, GC3 and GC4 (see Fig. 2) and large-scale groups, namely, GL1, GL2, and GL3 (see Fig. 3), by computing similar statistics for convective and stratiform precipitation as those computed for total precipitation earlier. Models of a given convective group show similarities in cloud model type, closure assumption, or trigger mechanism. For example, (i) in GC1, MIROC-ESM/ESM-CHEM, HadGEM2-CC/ES, ACCESS1-0, FGOALS-g2, and BCC-CSM1-1 use CAPE based closure, (ii) in GC2, GFDL-ESM2M/2G, NorESM1-M/ME and INMCM4 use CAPE based closure (GISS-E2H-R/R-CC, however, uses moisture convergence based closure), and most of them use CAPE based trigger, (iii) in GC3, most of the models use either CAPE or moisture convergence based closure and CAPE or moisture convergence based trigger (for more details see Table 2), and (iv) in GC4, CNRM-CM5/5-2 use moisture convergence based stability profile for closure, HadCM3, GFDL-CM3, EC-Earth use CAPE based closure, however, most of the models in this group use CAPE based trigger (for more details see Table 2).

Considering three major aspects of the convection parameterizaton schemes used in CMIP5 models, namely, (a) cloud model type, (b) closure, and (c) trigger, we find that: (1) based on cloud model type (spectral, bulk and mix), the distribution in GC1 is (2, 3, 2), in GC2 it is (2, 3, 3), in GC3 it is (2, 4, 6), and in GC4 it is (1, 4, 0), respectively, (2) based on closure mechanism (CAPE, moisture convergence, and other methods), the distribution in GC1 is (7, 0, 0), in GC2 it is (5, 3, 0), in GC3 it is (9, 3, 0), and in GC4 it is (3, 2, 0), respectively, and (3) based on trigger function (CAPE, moisture convergence, other methods), the distribution in GC1 is (5, 0, 2), in GC2 it is (4, 3, 1), in GC3 it is (5, 4, 3), and in GC4 it is (3, 2, 0), respectively. Further, we notice that some models in GC2 use moisture convergence for both trigger as well as closure, whereas, some models in GC3 use moisture convergence for triggering deep convection (similar to GC2) but for closure they use CAPE (for more details see Table 2). We also find that the cloud model type used in the convection parameterization schemes has very limited effect on simulated convective precipitation. For example, FGOALS-g2 and CESM-CAM5, GFDL-CM3 and MIROC-5 model pairs have a common cloud model type but do not show much similarity in their convective precipitation fields. This finding is in line with Yanai *et al*.^28^, wherein it was reported that for tropical convection both spectral and bulk methods were found to produce similar total vertical mass fluxes. Unlike the convective groups, which show large inter-group variations in convective precipitation, the large-scale groups do not show as much variations, likely due to lesser degree of differences in the large-scale precipitation parameterization schemes.

#### Convective Precipitation Biases in South Asian Summer Monsoon Simulations

**Figure 4:**
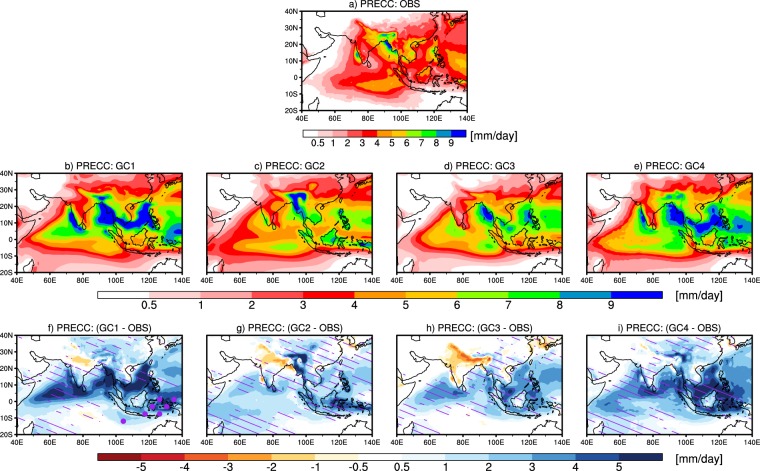
The spatial variation of mean JJAS (June–September) convective precipitation over the south Asian region from observation (**a**), GC1 (**b**), GC2 (**c**), GC3 (**d**), and GC4 (**e**). The biases in mean JJAS convective precipitation for different groups with respect to observation are shown in (**f**) for GC1, (**g**) for GC2, (**h**) for GC3, and (**i**) for GC4. Hatching show bias to be coming from atmospheric component and stippling show bias to be coming from oceanic components (i.e. biases in SSTs and atmosphere-ocean feedbacks) and the biases are significant the level of 99%.

Figure 4 shows spatial variation of mean seasonal convective precipitation and convective precipitation biases from observation in various convective groups. Observed convective precipitation is found to be highest over Indo-Burmese Mountain, Western Ghats (WG), moderate over central India and eastern equatorial Indian Ocean, and lowest over northwest India (Fig. 4a). In GC1, the convective precipitation is found to be highest over Indo-Burmese mountain, eastern Bay of Bengal (BoB), south Arabian Sea (AS) adjoining to WG, and south China Sea (SCS) (Fig. 4b), in GC2, the convective precipitation is found to be high only over northeast India (Fig. 4c), in GC3, convective precipitation is found to be high only over the Indo-Burmese mountain (Fig. 4d), and in GC4, convective precipitation is found to be high over eastern BoB, WG, and central SCS (Fig. 4e). Thus, in GC1, we find large significant overestimation over northern AS, Indo-Burmese mountain, and SCS (Fig. 4f), in GC2, we find large significant overestimation over northeast India (Fig. 4g), in GC3, we find small overestimation over entire south Asian region (Fig. 4h), and in GC4, we find the large significant overestimation over SCS, Indo-Burmese Mountain and eastern BoB (Fig. 4i). Small underestimation in convective precipitation is found over the Indo-Gangetic region in all convective groups except in GC4, with increase in spatial extent of the negative bias from GC1 to GC3. We also notice that model grouping and spatial pattern of convective precipitation biases do not change much irrespective of the changes in observational dataset type (for example, when we change PERSIANN-CDR data with GPCP data, convective model grouping and spatial pattern biases do not change much; figure not shown).

**Figure 5:**
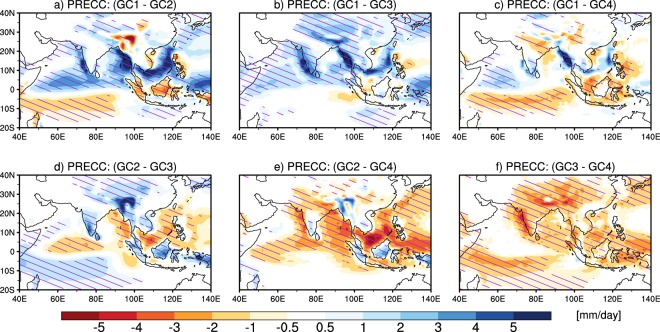
The differences in mean JJAS convective precipitation of each cluster with the other clusters: (**a**) GC1 and GC2, (**b**) GC1 and GC3, (**c**) GC1 and GC4, (**d**) GC2 and GC3, (**e**) GC2 and GC4, and (**f**) GC3 and GC4. Regions with differences that are statistically significant at 99% are hatched.

Some of the past studies have reported that large monsoon precipitation biases over the AS, Indian land, and Indo-Burmese mountains could be due to the cold Arabian Sea SST biases^15,29^. In another relevant study Levine and Turner^30^ have also shown this using numerical experiments that cool AS SST can delay south Asian summer monsoon and subsequently reduce monsoon precipitation. Next, we investigate how important are SST biases and atmosphere-ocean feedbacks in the convective precipitation biases discussed above, by analyzing the differences in biases in CMIP5 models with their corresponding AMIP5 counterparts. Hatching (in Fig. 4) indicates that the biases are primarily due to the atmospheric component, whereas, stippling indicates that the errors in SSTs and the atmosphere-ocean feedbacks are also important. Over a given grid point if the root mean square error (RMSE) in a given model group from AMIP5 simulations is greater than or equal to 80% of RMSE in the same model group from CMIP5 simulations the grid point is hatched, whereas, if the RMSE in the AMIP5 simulations is smaller than 20% of the RMSE in the corresponding CMIP5 simulations the grid point is stippled. In addition, we also impose a second condition of two-tailed student-t test for significant bias at 99% on hatching and stippling along with the first condition mentioned above. If a grid point is neither hatched nor stippled it means that the bias is either not significant or is due to both atmospheric and oceanic components. Thus, in all the convective groups, overestimation in convective precipitation over majority of the South Asian region seems primarily to be coming from the atmospheric component. Notably, the significant biases in GC1 models over the maritime continent seem to be coming from the SST biases (since the CMIP5 biases are found to be much higher than the AMIP5 biases). In order to further confirm that the model groups are distinctively different from each other, we analysed the inter-group differences (shown in Fig. 5), and find that there are indeed significant differences between the groups, thus also confirming the robustness of the method used for clustering.

**Figure 6:**
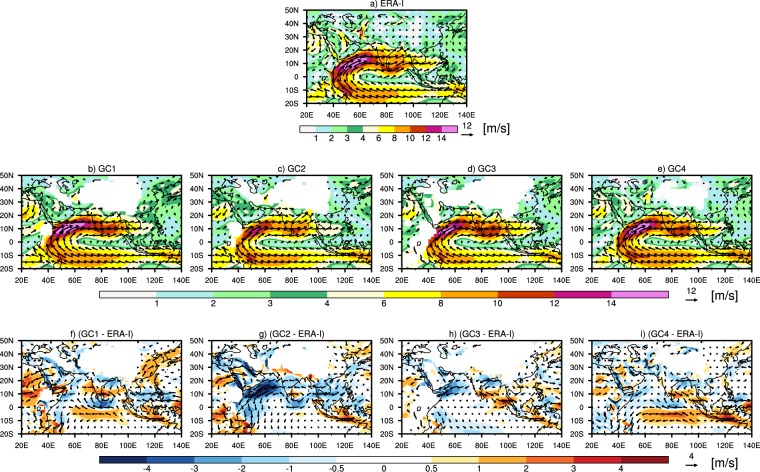
The spatial variation of mean JJAS (June – September) 850 hPa wind pattern over the south Asian region from (**a**) ERA-I, (**b**) models in convective group GC1, (**c**) GC2, (**d**) GC3, and (**e**) GC4. The biases in mean JJAS 850 hPa wind pattern for different convective groups with respect to reanalysis are shown in (**f**) for GC1, (**g**) for GC2, (**h**) for GC3, and (**i**) for GC4.

Figure 6 shows the spatial variation of mean wind pattern at 850 hPa from ERA-I and from the various convective groups (i.e. GC1, GC2, GC3 and GC4). Also shown are the corresponding biases for each of the groups. ERA-I shows a well-established cross-equatorial flow and well-established Somali current over northern AS, southern peninsular India and over the northern BoB (Fig. 6a) as reported in the literature^21,31^. The cross-equatorial current and Somali current are also found in all convective groups, however with varying magnitudes (Fig. 6b–e). From the mean wind biases: (a) in GC1 we find a very large cyclonic anomaly over the central equatorial Indian Ocean consistent with the overestimation in precipitation over eastern AS and BoB, easterly wind anomaly over western coast of AS, and consistent with the underestimation in precipitation over northern Indian region^31–33^; easterly wind anomaly over SCS consistent with the overestimation in precipitation over SCS^34,35^ (Fig. 6f), (b) in GC2 we find easterly wind anomaly to be low over peninsular India, high over AS and small over SCS, which is thus consistent with the underestimation in precipitation over northern Indian region, and overestimation over SCS. Weak cyclonic anomaly over central equatorial Indian Ocean (IO) is consistent with small overestimation in precipitation over equatorial IO, BoB, and eastern AS (Fig. 6g)^33^, (c) in GC3 we find the easterly wind anomaly over the western AS, northern BoB and westerly wind anomaly over peninsular India consistent with the large underestimation in convective precipitation over Indian region and small overestimation over rest of the domain, in line with previous studies^35,36^ (Fig. 6h), and (d) in GC4 we find the large easterly equatorial wind anomaly and westerly wind anomaly over central BoB causing more overestimation in precipitation over equatorial region, BoB and SCS, in line with previous studies^31,35^ (Fig. 6i).

#### Large-Scale Precipitation Biases in South Asian Summer Monsoon Simulations

**Figure 7:**
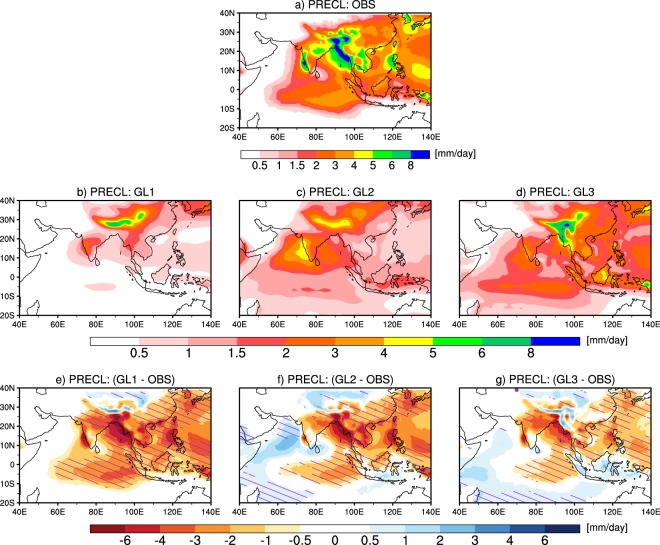
Same as Fig. 4 but for large-scale precipitation from observation and from large-scale precipitation groups (GL1, GL2, and GL3).

**Figure 8:**
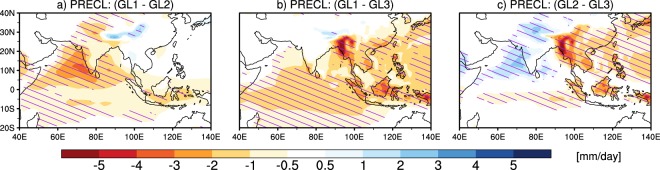
Same as Fig. 5, but for large-scale precipitation.

Figure 7 shows spatial variations of mean seasonal large-scale precipitation and large-scale precipitation biases from observations in various large-scale groups. It can be seen from Fig. 7a that observed large-scale precipitation is high over Indo-Burmese mountain, WG, and northeast India. In GL1, highest values are found over Himalayan foothills (Fig. 7b); in GL2, highest values are found over Himalayan foothills, WG and eastern Arabian Sea (Fig. 7c); in GL3, highest values are found over Himalayan foothills and over northeast India (Fig. 7d). From the bias patterns, GL1 shows large underestimation over Indo-Burmese mountain, eastern BoB, and WG (Fig. 7e), GL2 shows large underestimation over Indo-Burmese mountain and eastern BoB (Fig. 7f), and GL3 shows negative biases in line with GL1 and GL2 but with lower magnitudes (Fig. 7g). All large-scale precipitation groups also show the underestimation over central India and eastern equatorial Indian Ocean. In all the large-scale groups, underestimation in large-scale precipitation over majority of the south Asian region seems primarily to be coming from the atmospheric component (see hatching in Fig. 7). The underestimation in large-scale precipitation over WG in all large-scale groups seems to be due to both atmospheric and oceanic components. Similar to the convective precipitation grouping and spatial bias pattern, the large-scale model grouping and spatial bias pattern is also found to be minimally affected by the use of two different observational datasets (PERSIANN-CDR and GPCP; figure not shown). Similar to the analysis carried out to test the distinctiveness of the convective groups discussed above, we analysed the inter-group differences for the large-scale-precipitation-based groups (shown in Fig. 8), and find that the inter-group differences are significant.

## Conclusions

We have carried out a systematic analysis of the structure of precipitation biases in 33 CMIP5 and AMIP5 models, and have grouped them based on the correlation of their biases in total, convective and large-scale precipitation on global scale. We found that the grouping of models is somewhat sensitive to the variable used, i.e., a given pair of models that fall in the same total precipitation bias group may not necessarily fall in the same convective or large-scale precipitation bias group.

By grouping the CMIP5 models based on their convective precipitation biases we find that the similarity in convective precipitation biases in a given group primarily comes from similarity in closure assumptions and trigger mechanisms, and to a lesser extent on the details of the cloud models used in the deep convection parameterization schemes of the models. By grouping the CMIP5 models based on their large-scale precipitation biases we find that the degree of similarity in large-scale precipitation biases among model groups was much higher than that seen in the corresponding convective precipitation biases (based on convective precipitation grouping). Over the south Asian domain, we find many biases that are common across the groups. In general, each of the convective groups show largely positive biases, whereas, each of the large-scale groups show largely negative biases over the south Asian region, with spatially varying magnitudes. We find that the spatial pattern of biases in the convective precipitation in various model groups have prominent signatures in the 850 hPa wind circulation biases as well.

In agreement with some prior studies^5,8^, we find that if 2 models have the same atmospheric component the degree of similarity in their global precipitation bias patterns is quite high, as compared to that if some other component(s) are similar but the atmospheric components (especially the convection scheme) are quite different. This finding highlights the primary role played by the atmospheric component of the model in governing precipitation biases. To investigate this further, we compare the corresponding model biases from CMIP5 and AMIP5 simulations, and conclude that, in general, the precipitation biases primarily depend on the atmospheric component of the models, and to a lesser extent on biases in SSTs or atmosphere-ocean feedbacks, at least on timescales of the current analysis. Notably, we find that there is only one model group wherein the ocean component is primarily responsible for the simulated convective precipitation biases (found over the maritime continent region).

As a first step towards eliminating a given bias in a model it is important to know how the bias structure in the model compares to other models in the same group and models in different groups. Thus, a more informed and efficient model development approach may be designed for achieving improved simulations of global and regional climate. Not to mention, the spatial resolution used in CMIP5 models is too coarse to resolve weather features such as fronts, atmospheric rivers, cyclone properties, and thus, weather resolving climate models would be required for improving the accuracy of simulations even further^37,38^.

## Data and Methodology

The historical simulation of monthly convective (PRECC) and total precipitation (PRECT) dataset from 33 CMIP5 and AMIP5 models^39^ were downloaded from the Earth System Grid Federation (ESGF; https://esgf-node.llnl.gov/). We use the r1i1p1 ensemble member for all CMIP5 and AMIP5 models, since some models of CMIP5 and AMIP5 do not provide the individual convective and large-scale components for other ensemble members. Observed monthly total precipitation for 23 years (1983–2005) are from PERSIANN-CDR dataset, which is a high-resolution (0.25° × 0.25°) long-term satellite and observation merged precipitation dataset, developed by the Centre for Hydrometeorology and Remote Sensing (CHRS) at University of California Irvine (https://chrsdata.eng.uci.edu/; Nguyen *et al*.^40^). The PERSIANN-CDR dataset is first bilinearly interpolated to the 0.5° × 0.5° grid of TRMM 3A12 (1998–2013; 0.5 × 0.5 degree)^41^, and then the corresponding convective and large-scale precipitation components are computed from total precipitation, by using Eq. (1). The large-scale precipitation (PRECL) dataset for observation and for model simulations are computed by subtracting the convective components from the total precipitation as shown in Eq. (2). Monthly zonal (u) and meridional (v) wind dataset (1983–2005) at 850 hPa are used from ECMWF (ERA-I) reanalysis (https://apps.ecmwf.int/datasets/). The domain used for our analysis is 0–360°E; 40°S–40°N.

1 $$PRECC\,(PERSIANN\_CDR)=PRECT(PERSIANN\_CDR)\ast (\,\frac{PRECC\,(TRMM)}{PRECT\,(TRMM)})$$

2 $$PRECL=(PRECT-PRECC)$$

To compute inter-model similarity between the CMIP5 models, we follow a similar method as that used by Pennell and Reichler^3^ and Knutti *et al*.^8^ which are widely accepted methods for model genealogy studies. Thus, we first compute the normalized bias (*e*_*n*,*m*_) in the mean annual total, convective, and large-scale precipitation for the CMIP5 models by using Eq. (3)^3^:

3 $$\begin{array}{c}{e}_{n,m}=({f}_{n,m}-{o}_{n})/{\sigma }_{n}\\ n=1,2,3,\,\ldots \,\mathrm{..},\,N;\,m=1,2,3,\ldots .\,,M\end{array}$$

where ‘N’ and ‘M’ are the total number of grid points and total number of models used for this study, symbol ‘o’, ‘f’, and ‘*σ*’ represent observation, model output, and observed standard deviation (*σ*) for any variable. For example, the normalized bias for a particular model (m) can be written from Eq. (3) as ${e}_{m}=({e}_{1,m},\,{e}_{2,m},\ldots \ldots ,{e}_{N-1,m},\,{e}_{N,m})$. Subsequently, we compute the common biain total, convective, and large-scale precipitation from the multi-model mean bias $(\bar{e})$by using Eq. (4)^3^.

4 $$\bar{e}=(\frac{1}{M})\sum _{m=1}^{m=M}{e}_{m}$$

The portion of multi-model mean bias $(\bar{e})$ corresponding to each individual model bias (i.e. $r=Cor.\,({e}_{m},\bar{e})$) is then subtracted from the corresponding model bias, to make the individual model biases more dissimilar from each other by using Eq. (5)^3^.

5 $${d}_{m}={e}_{m}-r\bar{e}$$

Finally, the level of inter-model similarity is computed by applying the Pearson sample linear cross-correlation between the model pair biases [Cor. (*d*_*m*1_, *d*_*m*2_)]. The hierarchical structure of CMIP5 models is constructed by converting the correlation matrix into distance matrix by using weighted pair-wise average distance method^3,42^.

## Data Availability

All the data used in this study is in public domain and can be freely downloaded.
